# Supplementary material for: NK cells eliminate Epstein-Barr virus bound to B cells through a specific antibody-mediated uptake
Source: PLoS Pathog. 2021 Aug 20;17(8):e1009868. doi: 10.1371/journal.ppat.1009868 (PMC8409624; doi:10.1371/journal.ppat.1009868)
Supplement: S1 Fig — (PDF) [file ppat.1009868.s001.pdf]

S1 Supplementary figure

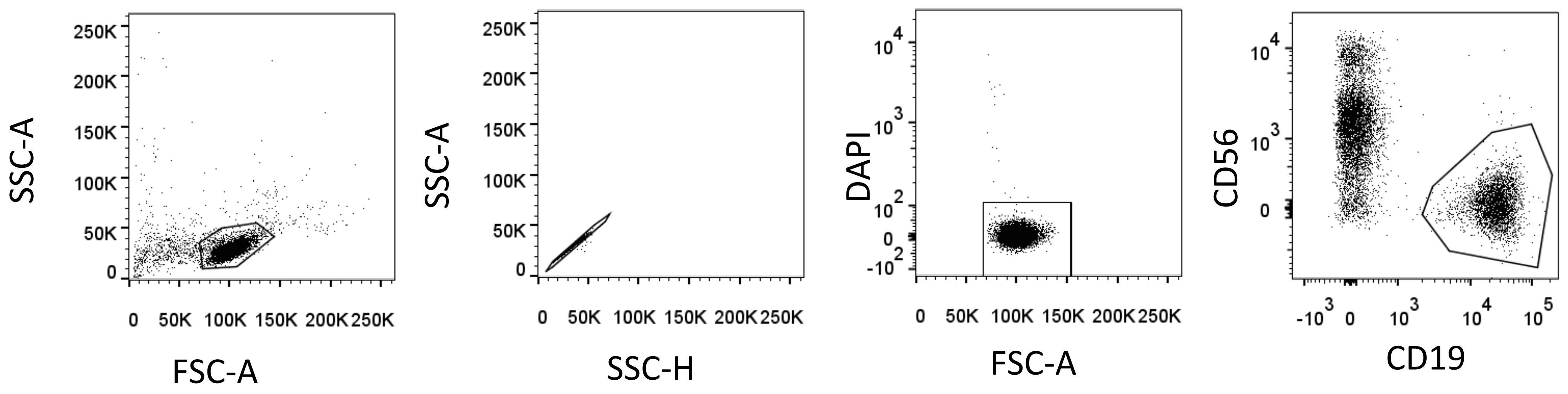

S1 Supplementary figure. Representative gating strategy for B cells in B-NK cell co-cultures.
