# Supplementary material for: NK cells eliminate Epstein-Barr virus bound to B cells through a specific antibody-mediated uptake
Source: PLoS Pathog. 2021 Aug 20;17(8):e1009868. doi: 10.1371/journal.ppat.1009868 (PMC8409624; doi:10.1371/journal.ppat.1009868)
Supplement: S2 Fig — (PDF) [file ppat.1009868.s002.pdf]

S2 Supplementary figure

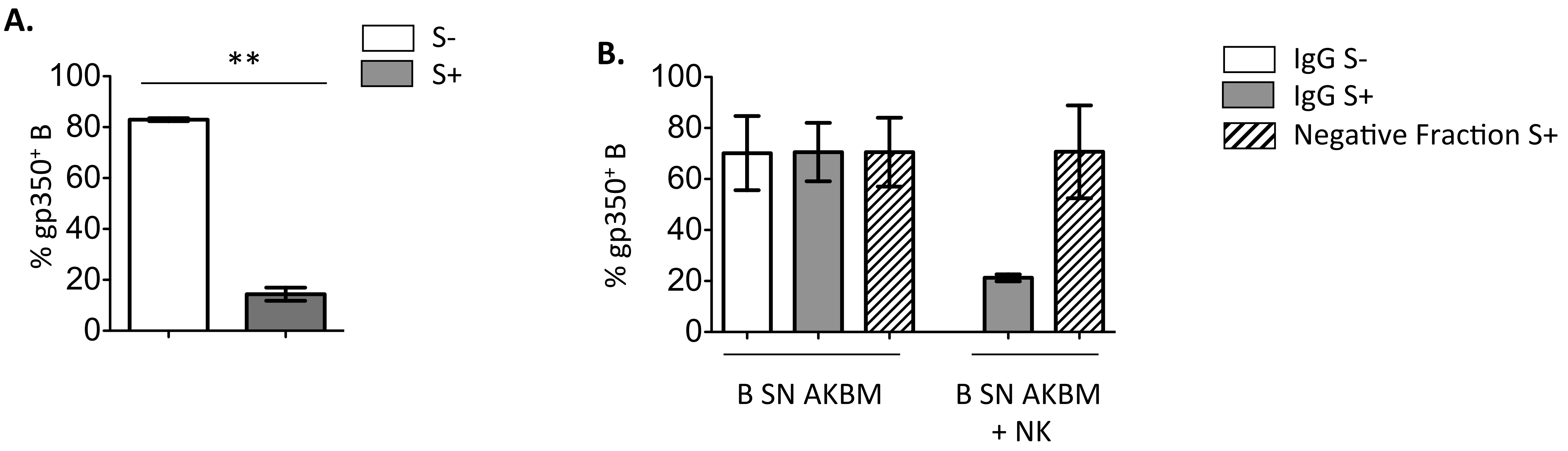

S2 Supplementary figure. Serum and purified serum IgG from EBV+ donors promotes NK cell-mediated elimination of viral particles coating B cells. (A, B) B cells were incubated with AKBM SN and stained for gp350. (**A**) B cells cultured with NK and 3% of serum from EBV S- (n=5) and EBV S+ (n=6) donors for 4h at 37°C. The percentage of gp350^{+} B was analyzed. Statistical analysis was performed with Mann-Whitney test. (**B**) B cells were cultured with or without NK cells, and with 0.45mg/ml IgG purified from EBV S- or S+ serum or the equivalent IgG-negative fraction of EBV S+ serum. The percentage of gp350^{+} B cells of two experiments is shown.
