# Supplementary material for: NK cells eliminate Epstein-Barr virus bound to B cells through a specific antibody-mediated uptake
Source: PLoS Pathog. 2021 Aug 20;17(8):e1009868. doi: 10.1371/journal.ppat.1009868 (PMC8409624; doi:10.1371/journal.ppat.1009868)
Supplement: S3 Fig — (PDF) [file ppat.1009868.s003.pdf]

S3 Supplementary figure

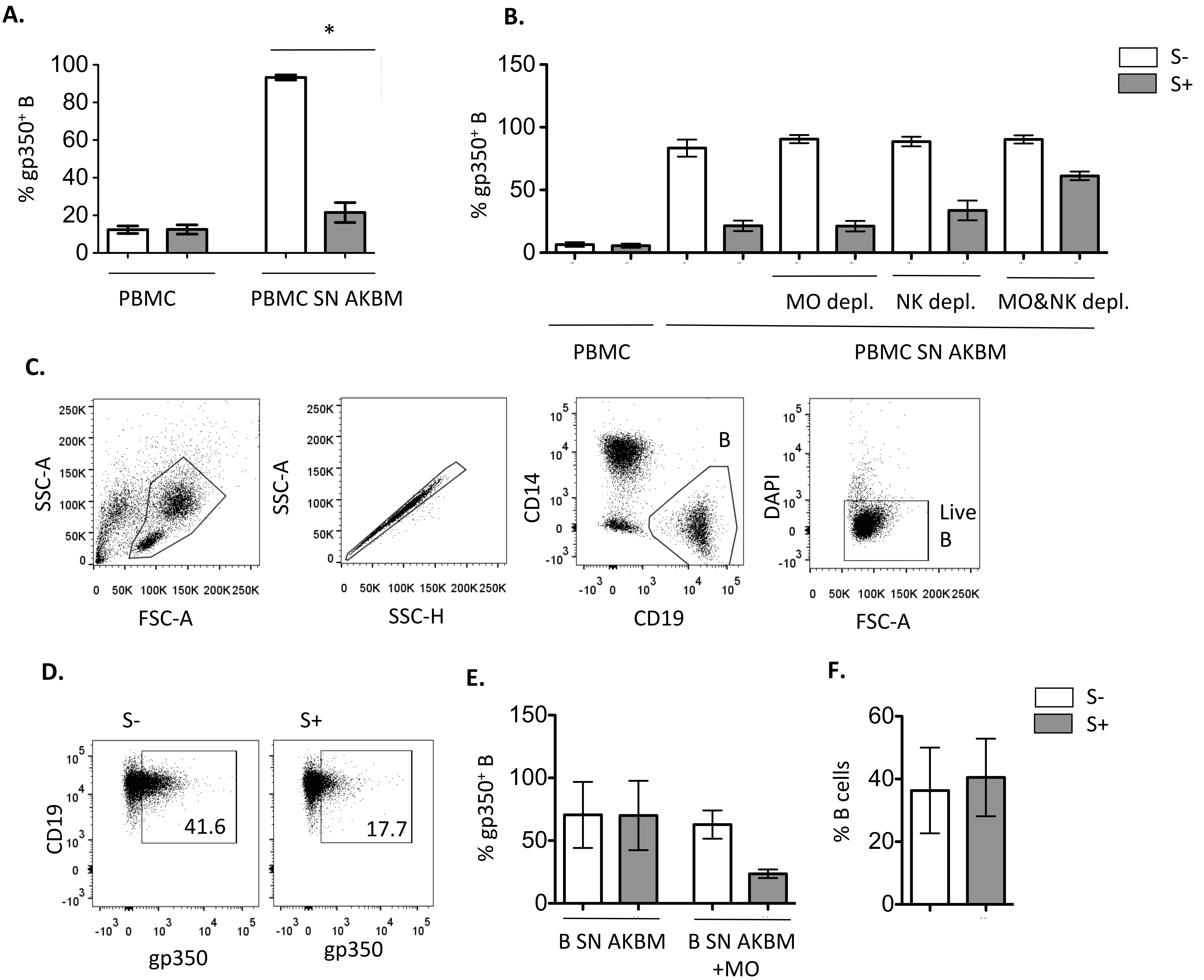

S3 Supplementary figure. Peripheral blood monocytes remove VP attached to B cells through anti-EBV Abs. (**A**) PBMC were incubated with AKBM SN, stained for gp350, cultured with EBV S- or S+ serum for 4h and analyzed for gp350 staining on B cells (n=5). Wilcoxon test was applied to the last two groups. (**B**) PBMC depleted or not of monocytes (MO), NK cells or both were incubated with AKBM SN, washed, stained for gp350, cultured with EBV S- or S+ serum for 4h and analyzed for gp350 staining on B cells (n=3). (**C-F**) B cells were incubated with AKBM SN, washed, stained for gp350, and co-cultured alone or with purified autologous MO at a 1:1 ratio in the presence of EBV S- or S+ serum. (**C-D**) Representative gating strategy and Gp350 expression on B cells. (**E-F**) Results of three different experiments and % B cells detected after co-cultures.
