# Supplementary material for: NK cells eliminate Epstein-Barr virus bound to B cells through a specific antibody-mediated uptake
Source: PLoS Pathog. 2021 Aug 20;17(8):e1009868. doi: 10.1371/journal.ppat.1009868 (PMC8409624; doi:10.1371/journal.ppat.1009868)
Supplement: S4 Fig — (PDF) [file ppat.1009868.s004.pdf]

S4 Supplementary figure

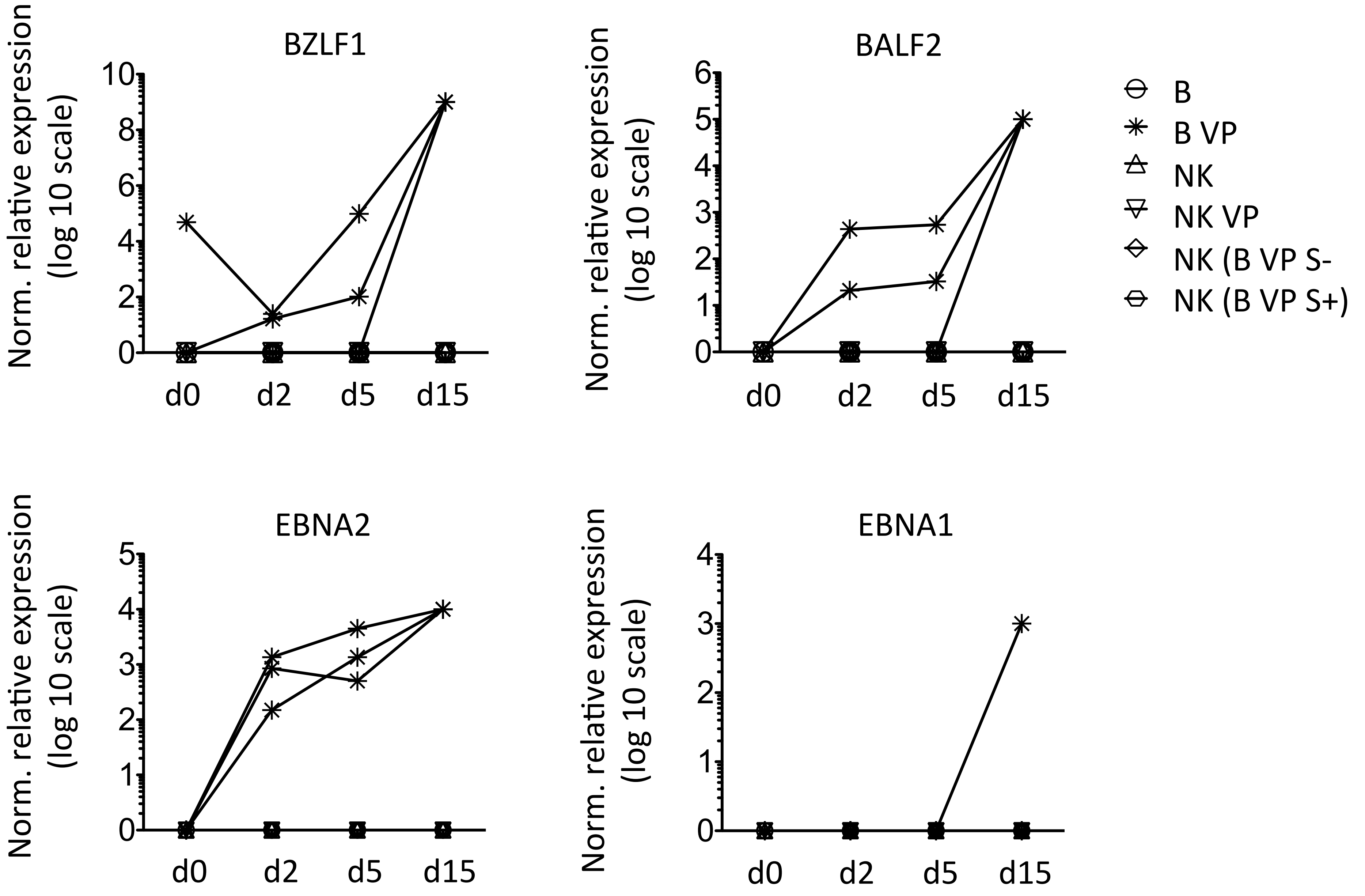

S4 Supplementary figure. Undetectable EBV infection in NK cells co-cultured with B95.8-coated B cells and anti-EBV Abs. B cells were incubated with B95.8 VP for 1h at 4°C, washed and co-cultured with NK cell and EBV S- or S+ serum for 4h. NK cells were purified by negative selection and cultured for the indicated time (d: days), in parallel with control B and NK cells which had been incubated or not with B95.8 VP for 1h at 4°C and washed. Samples were analyzed for RNA of the indicated EBV genes and of β2-microglobulin as internal control by quantitative nested PCR (for BZLF1, BALF2 and EBNA2) or quantitative PCR (EBNA1). The relative expression to β2-microglobulin was normalized to the maximum value of each experiment allowing to display the whole range of values in a log 10 scale. For intuitive data representation, non-detects were given a value of 1. Results of three experiments individually plotted are displayed for each gene.
