# Supplementary material for: NK cells eliminate Epstein-Barr virus bound to B cells through a specific antibody-mediated uptake
Source: PLoS Pathog. 2021 Aug 20;17(8):e1009868. doi: 10.1371/journal.ppat.1009868 (PMC8409624; doi:10.1371/journal.ppat.1009868)
Supplement: S5 Fig — (PDF) [file ppat.1009868.s005.pdf]

S7 Supplementary figure

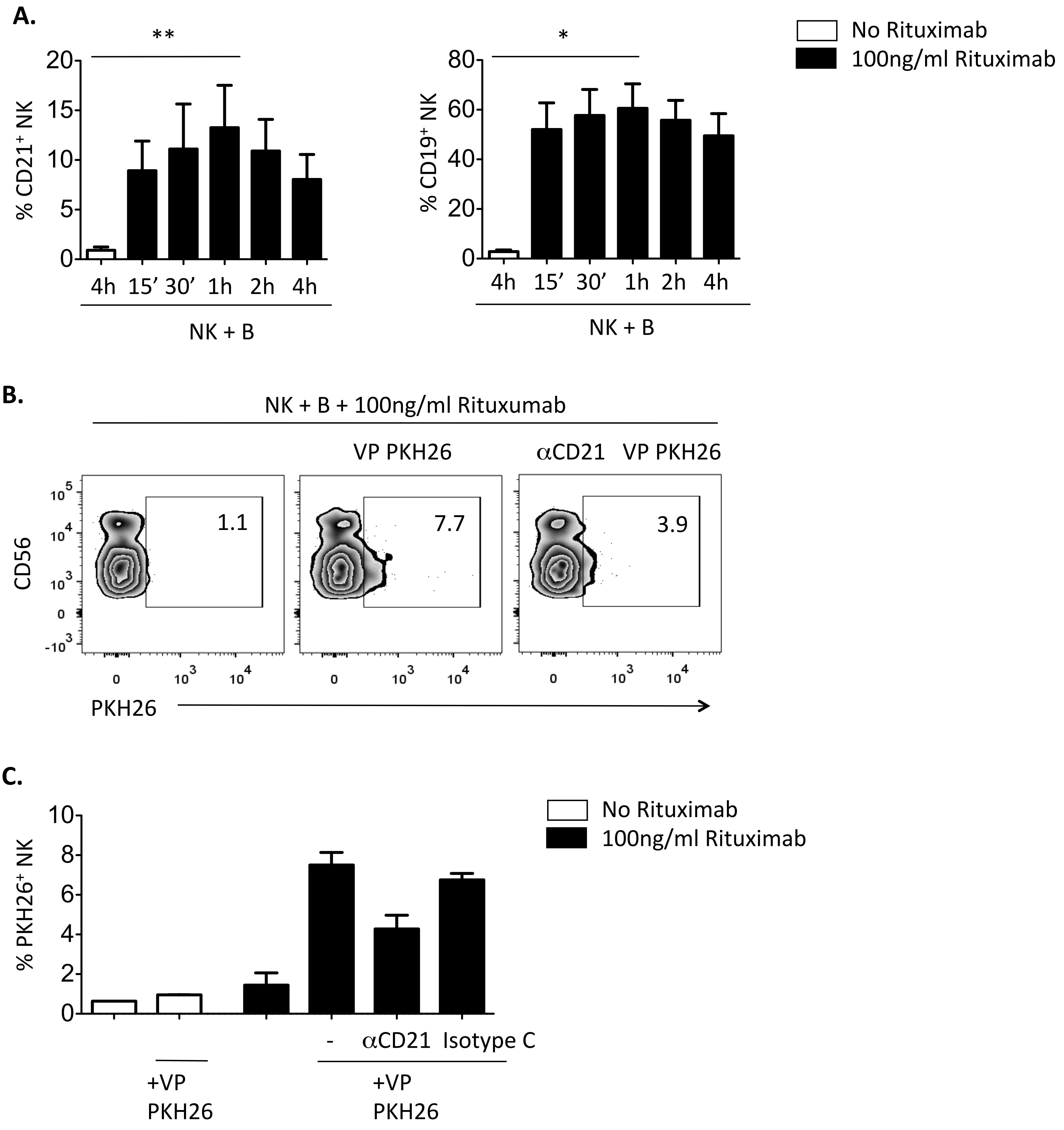

S7 Supplementary figure. CD21 acquired by NK cells through Ab-induced trogocytosis can interact with EBV VP. (**A**) NK and B cells were co-cultured for the indicated times in the presence of 100ng/ml of Rituximab analyzing CD21 and CD19 on NK cells (n=4). Statistical analysis was performed with Friedman test with Dunn’s post-test. (**B-C**) NK and B cells were co-cultured for 1h in the presence of 100ng/ml of Rituximab and treated or not with 20μg/ml of blocking anti-CD21 Ab or isotype control Ab for 30’ at 4°C. Samples were incubated with PKH26-stained B95.8 VP for 2h at 4°C, washed and analyzed for PKH26 binding to NK cells. Representative plots of the indicated conditions (**B**) and results of two different experiments (**C**) are shown.
