# Supplementary material for: NK cells eliminate Epstein-Barr virus bound to B cells through a specific antibody-mediated uptake
Source: PLoS Pathog. 2021 Aug 20;17(8):e1009868. doi: 10.1371/journal.ppat.1009868 (PMC8409624; doi:10.1371/journal.ppat.1009868)
Supplement: Attachment — Submitted filename: Response to reviewers 120721.docx [file ppat.1009868.s008.docx]

Point-by-point response to reviewers

*Reviewer #1: 1. The authors suggest that the observed mechanism might be a new effector function of NK cells, but it remains unclear, if similar effects could be observed for all Fc receptor expressing cells. Are NK cells better than monocytes in removing EBV particles from B cell surfaces? NK cells should be compared in this respect with some classical phagocyte population.*

To investigate whether other FcgR-expressing cells can also eliminate B cell-bound EBV VP through anti-EBV Abs, we assessed the effect of depleting monocytes, NK cells or both on the ability of PBMCs to remove VP from B cells. In these experiments, only depletion of both cell populations substantially inhibited VP elimination, pointing out a role for monocytes. This was directly confirmed in experiments co-culturing VP-coated B cells with purified monocytes which in the presence of S+ serum reduced the VP-associated signal on B cells. These preliminary data support that, as anticipated in the original version, other FcgR-expressing cells can also remove B cell-bound VP. Data are shown in Supplementary figure 3 and mentioned in Results (page 7, lines 134-143), Discussion (page 15, lines 319-321) Methods (page 20, lines 424-428; page 22, line 493) and Supplementary figure captions (page 38, lines 932-933)

*2. Chronic active EBV infection (CAEBV) often spreads to other lymphocytes than B cells including NK cells. Could the observed mechanism facilitate NK cell infection by EBV? Any sign of latent EBV gene expression in NK cells after the antibody mediated uptake? As an alternative to latent EBV gene expression, GFP expression from recombinant EBV could be monitored in NK cells that have taken up EBV particles.*

To address the reviewer´s remark, we assessed EBV infection of NK cells after co-culture with EBV VP-coated B cells and EBV S+ serum by measuring expression of four EBV genes (BZLF1, BALF1, EBNA1 and EBNA2), three of which (BZLF1, BALF1 and EBNA1) have been described to be expressed by in vitro-EBV-infected NK cell lines (new Ref. 50). RNA was measured in NK cells purified after the co-culture, straight away or after culturing them for 2, 5 or 15 days; in parallel, controls were set up culturing B or NK cells with or without EBV VP. As shown in Supplementary figure 4, though transcription of these genes was detectable at some point in EBV-treated B cells, we were unable to detect their expression in primary NK cells. In the revised version these experiments are described in Results (page 10, line 202-204, 206), Discussion (page 17, lines 354-363), Materials and Methods (page 21, lines 454-459; page 23, lines 500, 511-518), References (page 31, lines 748-750) and Supplementary figure captions (page 38, lines 934-935).

*3. The authors observed CD19, CD20 and CD21 transfer to NK cells by trogocytosis. Is this of any functional consequence? Can CD21 expression and MHC class II up-regulation on activated NK cells be used for EBV infection? Is such infection blocked* by antibodies against these respective surface markers?

To assess whether CD21 transferred to NK cells might interact with EBV VP avoiding the putative interference of B cell-bound VP involved with the Ab-mediated trogocytosis process, we took advantage of a report describing CD20 trogocytosis by NK cells upon ADCC against Rituximab treated B cells [35]. These observations were reproduced detecting also CD21 on NK cells (S7 Suppl. Fig. A) at levels greater than those observed in our experimental system with VP-coated B cells, thus potentially increasing the sensitivity of the VP-binding assay. Under these conditions binding of PKH26-labeled VP was detectable on the surface of NK cells, and was partially blocked by anti-CD21 mAb (S7 Suppl. Fig. B). These experiments are described in Results (page 12, lines 251-261), Discussion (page 16, lines 351-353), References (page 30, lines 702-705), Materials and Methods (page 21, lines 449- 451) and Supporting information captions (page 38, lines 938-939) of the revised version.

*Reviewer #2: 1. For flow cytometric analysis (Figures 2, 5, 6, 7 ) the authors should demonstrate with some rigour (Other than comparison with the Rituximab control) how they can exclude that a small proportion of transient B-NK cell conjugates are not being detected within the NK cell gate thereby detecting B cell markers CD19, CD20 and CD21 and also virus gp350 labelled B cells within this gate.
2. How are small populations of dead cells being excluded from the co-culture systems?*

Though not specified in the original manuscript, cell conjugates were excluded by SSC-H vs SSC-H gating, and dead cells were excluded by DAPI staining. In the revised version new S1 Supplementary figure illustrates the gating strategy described in Results (page 7, line 125-126), Materials and Methods (page 20, lines 445-446; page 21, line 447) and Supporting information captions (page 37, lines 928-929).

*3. Fig 7. Some clarification is needed for the data in figure 7A and 7B. In 7A, the right hand plot suggests a gradual attrition of virus from B cells with or without immune sera in the absence of NK cells. Does the co-culture system not simply reflect an acceleration of the process? The attrition of S+ in the absence of NK cells implies that this process is, at least in part, passive. Significance values should also be added to these plots to compare values across the time frame and between S- and S+ data points.*

As noticed by the reviewer, detection of VP was reduced when staining with the PE-labeled anti Ig antibody was carried out after 4h culture (Fig. 7A right) as compared to staining at the beginning (Fig. 7A left). This is attributable to the parallel VP internalization process by B cells, which was not specified in the text and has been addressed in the revised version. When NK cells and S+ serum are present, the Ab-mediated uptake rate is faster than VP internalization by B cells, which lose the VP-associated signal even if staining with the PE-labeled anti Ig Ab has been performed before the co-culture, as done in most experiments. For a clear comparative perception of both processes, a new panel has been added in Figure 7 showing the results of PE MFI of B cells in the presence of NK cells normalized to that detected at time 0 of B cells alone. Comments have been added in Results (page 12, lines 265; page 13, lines 267-271), Material and Methods (page 26, lines 571-573) and Figure captions (page 36, line 904; page 37, lines 907-908, 910).

*4. Figure 7D and E and supplementary video microscopy data. Although the this data does support uptake of viral components, what is the cumulative frequency of PKH26+ NK cells detected in these systems at any given point across the entire time frame and how does this compare to the steady state levels detected by flow cytometry (See point 1, above)*.

Microscopy experiments were carried out for a qualitative visual assessment of the process. As compared to flow cytometry, less cells could be evaluated and differences in the design of the experimental systems render difficult a precise quantitative comparison of the corresponding results obtained. For instance, in flow cytometry experiments 15 min was the minimum time during which cells were in culture in a U bottom 96 well plate at 37ºC, while in microscopy experiments cells were subsequently attached to poly-L-lysine-coated coverslips for additional 15 min at 37ºC. In video microscopy experiments, cells were allowed to settle for 5 min and cultured on a flat-bottom plate, thus decreasing cell contacts and consequently reducing the process efficiency.

*5. The authors demonstrate the co- localisation of EBV associated components with NK cell endosomal and lysosomal markers. Can they exclude the persistence of viable viral particles?
This point should also be discussed in the context of evidence for or against a role for antibody facilitating active infection of NK cells.*

To address the reviewer´s remark, we assessed EBV infection of NK cells after co-culture with EBV VP-coated B cells and EBV S+ serum by measuring expression of four EBV genes (BZLF1, BALF1, EBNA1 and EBNA2), three of which (BZLF1, BALF1 and EBNA1) have been described to be expressed by in vitro-EBV-infected NK cell lines (new Ref. 50). RNA was measured in NK cells purified after the co-culture, straight away or after culturing them for 2, 5 or 15 days; in parallel, controls were set up culturing B or NK cells with or without EBV VP. As shown in Supplementary figure 4, though transcription of these genes was detectable at some point in EBV-treated B cells, we were unable to detect their expression in primary NK cells. In the revised version these experiments are described in Results (page 10, line 202-204, 206), Discussion (page 17, lines 354-363), Materials and Methods (page 21, lines 454-459; page 23, lines 500, 511-518), References (page 31, lines 748-750) and Supplementary figure captions (page 38, lines 934-935).

***Part III – Minor Issues: Editorial and Data Presentation Modifications*** *Please use this section for editorial suggestions as well as relatively minor modifications of existing data that would enhance clarity.*

*Reviewer #1: 1. The authors suggest that uncontrolled EBV infection in CD16A deficient patients points towards the importance of the described mechanism for EBV specific immune control in vivo. However, they should acknowledge that it was proposed that CD16A is required for CD2 function on NK cells outside of ADCC.*

We believe that the reviewer refers to the publication by Grier et al. (new ref 51). The reference has been added in References (page 31, lines 751-753) and commented in Discussion (page 17, lines 365-369).

*Reviewer #2: 1. Fig 6. Could this be re-ordered to separate the subplots according to experimental conditions and to label the culture conditions more clearly.*

Culture conditions have been labeled in Figure 6.
